# Age-specific effects of structural and functional connectivity in prefrontal-amygdala circuitry in women with bipolar disorder

**DOI:** 10.1186/s12888-018-1732-9

**Published:** 2018-06-05

**Authors:** Yanqing Tang, Yinzhu Ma, Xuemei Chen, Xuesheng Fan, Xiaowei Jiang, Yifang Zhou, Fei Wang, Shengnan Wei

**Affiliations:** 1 Brain Function Research Section, Department of Radiology, First Affiliated Hospital, China Medical University, 155 Nanjing North Street, Shenyang, 110001 Liaoning People’s Republic of China; 2 Department of Psychiatry, First Affiliated Hospital, China Medical University, 155 Nanjing North Street, Shenyang, 110001 Liaoning People’s Republic of China; 3 Department of Geriatric Medicine, First Affiliated Hospital, China Medical University, Shenyang, Liaoning People’s Republic of China; 4 Department of Radiology, First Affiliated Hospital, China Medical University, Shenyang, Liaoning People’s Republic of China; 5 Department of Psychiatry, Yale University School of Medicine, New Haven, CT 06511 USA

**Keywords:** Bipolar disorder, Female, Age, Functional connectivity, Diffusion tensor imaging

## Abstract

**Background:**

Bipolar disorder (BD) is a serious mental illness. Several studies have shown that brain structure and function changes and the development of BD are associated with age and sex differences. Therefore, we hypothesized that the functional and structural neural circuitry of BD patients would differ according to age. The amygdala and prefrontal cortex (PFC) are play a key role in the emotional and cognitive processing of patients with BD. In this study, we used magnetic resonance imaging (MRI) to examine the structural and functional connectivity within amygdala-PFC neural circuitry in women with BD at different ages.

**Methods:**

Forty-nine female patients with BD who were aged 13–25 years and 60 age-matched healthy control (HC) individuals, as well as 43 female patients with BD who were aged 26–45 years and 60 age-matched HC individuals underwent resting-state functional MRI (rs-fMRI) and diffusion tensor imaging to examine the structural and functional connectivity within the amygdala-PFC neural circuitry.

**Results:**

We found abnormalities in the amygdala-PFC functional connectivity in patients aged 13–25 years and significantly different fractional anisotropy (FA) values in patients aged 26–45 compared with the age-matched HCs. The significance of these findings was indicated by corrected *p* values of less than 0.05 (uncorrected *p* values less than 0.001).

**Conclusions:**

The findings in this cross-sectional study suggested that abnormalities in the functional connectivity of the amygdala-PFC neural circuitry are related to the pathophysiology of BD in women aged 13–25 years, while changes in the structural integrity of this neural circuitry are associated with the pathophysiology of BD in women aged 26–45 years. Therefore, functional and structural brain alterations may occur at different ages in female patients with BD.

**Electronic supplementary material:**

The online version of this article (10.1186/s12888-018-1732-9) contains supplementary material, which is available to authorized users.

## Background

The human brain is a complex circuit system, and the development of the brain involves marked changes during aging. Comparisons of the brain weights of people of different ages have suggested that considerable volume changes occur during brain development. A review of 56 longitudinal magnetic resonance imaging (MRI) studies involving several methods has indicated that the volume of the whole brain changes throughout life [1], while another review has shown that age-related changes in the white matter (WM) continue throughout childhood and adolescence [2]. Age has been shown to have global and large effects on the volumes of the cortex, WM, and subcortical structures. Furthermore, regionally selective and temporally heterochronological changes in the superficial WM magnetization transfer ratio with age have been demonstrated [3]. Similarly, functional connectivity (FC) MRI studies have documented obvious changes in the development of the human brain. A review of the development of human functional brain networks has shown using resting-state (rs)-functional MRI (rs-fMRI) that functional networks continue to develop from infancy through adolescence [4]. Additionally studies have suggested that the development of the human brain changes with aging [4, 5]. Several neuroimaging studies have shown that the development of the brains of patients with bipolar disorder (BD), which is a serious mental illness affecting approximately 1.5–3% of the general population [6], also changes with age [7–9]. Thus, a better understanding of these age-related brain changes in BD is critical to distinguish BD-specific brain development and healthy brain development.

Sex differences in brain structure and function have been noted throughout the life span [10–13]. Female brain development may differ from male brain development because of hormonal differences. These sexual differences in brain development have also been found in patients with BD [14–16]. A voxel-based morphometric and diffusion tensor imaging (DTI) study conducted only in male bipolar patients has reported that the patients had greater gray matter volumes in the left thalamus and bilateral basal ganglia as well as decreased fractional anisotropy (FA) values in the left posterior corona radiata [17]. However, few studies have investigated the changes that occur in the brains of female bipolar patients during development and aging. Investigations of female brain development are critical in order to better distinguish sexual differences and better understand the underlying pathophysiological mechanisms of BD. We hypothesized that BD patients of different ages would show differences in their functional and structural neural circuitry because of the brain changes associated with age and sex.

In particular, the amygdala and prefrontal cortex (PFC), which play a key role in emotional and cognitive processing, have been strongly implicated in BD [18–20]. Previous functional MRI analyses have provided convergent evidence of functional abnormalities from the amygdala to PFC in BD [21–28]. Additionally, the uncinate fasciculus (UF), which is an anterior WM structure that is critically important for the amygdala-ventral PFC connection [29], has also been implicated in emotional and cognitive processing [29–31]. Increasing evidence implicates abnormalities in the structural integrity of uncinate WM in BD [32–38].

A more sensitive technique to use to assess WM integrity is DTI, which provides quantitative information regarding water mobility through tissue. The most widely employed DTI index, FA, estimates the directionality and continuity of fiber tracts. Various methods of DTI data analysis have been developed based on this technique. A review of diffusion imaging studies of WM integrity in patients with BD compared to healthy controls (HCs) has shown that most studies have reported decreased FA values in specific brain regions, such as the UF [39]. However, the functional and structural connectivity of the brains of female BD patients of different ages has not been fully explored. In studies of neurological disorders, FC and DTI methods have been combined to show abnormalities in both the FC and WM connections between brain regions [40, 41]. Therefore, a combination of functional and structural connectivity techniques was applied herein to test the study hypothesis that amygdala-PFC functional connections and the structural integrity of amygdala-PFC WM connections, including the UF, which provides major WM connections between these structures, would differ between patients with BD and HCs [40, 41]. In this study, we combined DTI and rs-fMRI to examine the structural and FC of the amygdala-PFC neural circuitry in female patients with BD with age ranges of 13–25 and 26–45 compared with age-matched HCs. We selected the age of 25 as the boundary line between the groups because the human brain is thought to be fully mature at this age [5]. We hypothesized that the structural and FC between the amygdala and PFC would be altered in female patients with BD of different ages compared with HCs.

## Methods

### Participants

Forty-nine female patients with BD aged 13 to 25 and 60 age-matched HC individuals were recruited for this study; we also recruited 43 female patients with BD ages 26–45 and 60 age-matched HC participants. All participants of BD were identified from the outpatient clinics at the Department of Psychiatry of First Affiliated Hospital of China Medical University, Shengyang, China. We used advertisements to recruit the HC participants from the surrounding community. After a detailed description of the present study, all participants provided written informed consent as approved by the ethics committee of the Institutional Review Board of China Medical University. If their age were less than 18 years old, they and their parent/legal guardian also provided written informed consent. Inclusion criteria of this study for participants were: all BD participants met the Structured Clinical Interview for DSM-IV Axis I Disorders (aged > 18 years) or the Schedule for Affective Disorders and Schizophrenia for School-Age Children-present and Lifetime Version (younger than 18) criteria for Type I bipolar disorder, and they also did not have any other current or lifetime Axis I disorders, including alcohol and substance abuse or dependence; All HC participants and their first-degree relatives did not have any history of mental disease diagnosis measured by one of the above two diagnostic tools. In order to confirm and rule out mental disease diagnosis, all participants were independently interviewed by two trained psychiatrists. Severities of mood symptoms were evaluated in all subjects by the Hamilton Depression Rating Scale (HAMD), Young Manic Rating Scale (YMRS), and Hamilton Anxiety Rating Scale (HAMA).

### MRI data acquisition

MRI data was acquired at the First Affiliated Hospital of China Medical University using a GE MR Signa HDX 3.0 T MRI scanner with a standard 8-channel head coil as the date was obtained in our previous study [42]. We used foam pads to minimize head motion. All participants remained awake with their eyes closed for the duration of the scan. The fMRI data were acquired in parallel with the anterior–posterior commissure plane using a spin echo planar sequence. We used the following parameters: repetition time (TR) = 2000 ms; echo time (TE) = 40 ms; matrix = 64 × 64; field of view (FOV) = 24 × 24 cm^2^; 35 three-millimeter slices without gap; and scan time = 6 min 40 s.

### FC and DTI processing

FC and DTI data processing followed our prior studies [42, 43]. For the FC analysis, we performed a correlation between the amygdala as the seed region of interest (ROI) and all PFC voxels using REST. We defined the bilateral amygdala ROI using the automated anatomical labeling template [44]. In our study the PFC mask included Brodmann areas 9–12, 24, 25, 32, and 44–47 created using the normalized T1-weighted images of all subjects, which were first skull-stripped using BrainSuite2 (http://brainsuite.usc.edu).

DTI data were processed using the Pipeline for Analysing brain Diffusion images (PANDA) software [45] (version 1.2.3, http://www.nitrc.org/projects/panda/). At first, we must convert DICOM files to NIfTI images, estimate the brain mask, crop the images, and correct for eddy-current effects. Next, we need to average the acquisitions and calculated DTI metrics such as fractional anisotropy and mean diffusivity for statistical analysis. Individual diffusion metric images were transformed to Montreal Neurological Institute (MNI) space using spatial normalization with 1 mm^3^ voxels. We used the ICBM-DTI-81 WM labels for parcellation of all WM into ROIs [46]. and calculated regional diffusion metrics by averaging the values within each region of the WM atlas using PANDA. We selected the UF as our ROI in the present study.

### Statistical analysis

Two-sample *t*-tests were conducted to compare the demographic data and HAMD, YMRS, HAMA scores and rs FC between the 2 groups of patients (*p* < 0.05) and group differences in the FA values in the UF (*p* < 0.05) using SPSS (IBM Corporation, Armonk, NY, USA). The DTI and FC differences were separately set at corrected *p* < 0.05 (uncorrected *p* < 0.001) using AlphaSim correction (performed using DPABI_V1.2_141101 software, http://rfmri.org/dpabi).

## Results

### Demographic and clinical characteristics

This study recruited 49 female patients with BD who were aged 13–25 years and 60 age-matched HC individuals as well as 43 female patients with BD aged 26–45 years and 60 age-matched HC individuals. The demographics and clinical characteristics of the female participants are shown in Table 1. No significant differences were observed in age (*p* = 0.58, *p* = 0.33) between the total BD and HC groups aged 13–25 and 26–45 years. Compared to the HCs, the participants with BD had significantly greater levels of depression, mania, and/or anxiety, which were measured by HAMD, YMRS, and HAMA (*p* < 0.001).

**Table 1:** Demographic and clinical characteristics of the female participants

	Female participants aged 13 to 25				Female participants aged 26 to 45			
Variable	BD (*n* = 49)	HC (*n* = 60)	*T/χ* ^*2*^	*p*	BD (*n* = 43)	HC (*n* = 60)	*T/χ* ^*2*^	*p*
Age (years)	19.90(3.32)	20.27(3.51)	−0.56	0.58	32.51(5.31)	33.75(6.87)	−.99	0.33
Race			2.24	0.14			0.77	0.38
Han	34(69.39)	49(81.67)			38(88.37)	56(93.33)		
Minority	15(30.61)	11(18.33)			5(11.63)	4(6.67)		
Education (years)	12.37(2.74)	13.59(2.67)	0.56	0.58	14.10(3.23)	14.97(3.32)	1.29	0.20
HAMD	11.06(9.18)	1.44(1.62)	7.09	.000	12.57(11.69)	0.96(1.68)	6.39	.000
YMRS	7.48(10.34)	0.21(0.89)	4.86	.000	12.57(11.69)	0.31(0.96)	3.93	.000
HAMA	8.51(8.98)	0.91(1.56)	5.61	.000	9.24(10.51)	1.07(2.14)	4.90	.000
State								
Depressed	23(46.94)	–	–	–	20(46.51)	–	–	–
Manic	14(28.57)	–	–	–	5(11.63)	–	–	–
Stable	12(24.49)	–	–	–	18(41.86)	–	–	–
First episode, yes	31(63.27)	–	–	–	14(32.56)	–	–	–
Medication, yes	32(65.30)	–	–	–	29(67.44)	–	–	–
Duration (month)	22.06(23.24)	–	–	–	70.26(78.32)	–	–	–

Data are *n* (%) or mean (SD). *BD* bipolar disorder, *HC* Healthy controls, *SD* Standard Deviation, *HAMD* Hamilton Depression Rating Scale, *BPRS* Brief Psychiatric Rating Scale, *HAMA* Hamilton Anxiety Rating Scale

### FC and DTI results

**Fig. 1:**
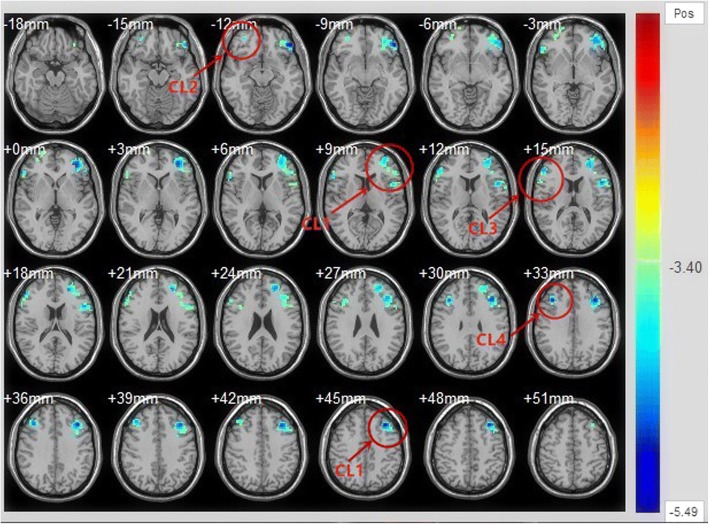
Results of two-sample *t*-tests showing abnormalities in the resting-state functional connectivity of the amygdala-prefrontal cortex (PFC) circuit in patients with bipolar disorder (BD) compared with healthy controls aged 13–25 years. The significance of these findings corresponded to a corrected *p* value of < 0.05 (uncorrected *p* value of < 0.001)

We found 4 PFC regions that were significantly different between HC and participants with BD aged 13–25 years (Table 2; Fig. 1). The rs FC of the amygdala-PFC did not differ significantly between HCs and BD patients aged 26–45 years. These findings corresponded to a corrected *p* < 0.05 (uncorrected *p* < 0.001).

**Table 2:** Bilateral amygdala showing significant changes in functional connectivity between patients with bipolar disorder (BD) and healthy controls aged 13–25 years

	MNI Coordinates				
Cortical Regions	Cluster Size	X	Y	Z	*T* values^1^
CL1_Ventral and dorsal prefrontal cortex	903	−51	42	−12	− 5.49
CL2_Ventral prefrontal cortex	47	33	51	−12	−4.11
CL3_Dorsal lateral prefrontal cortex	105	54	36	15	−4.39
CL4_Dorsal lateral prefrontal cortex	94	39	27	33	−5.04

*CL* cluster; ^1^These findings correspond to a corrected *P* < 0.05

**Fig. 2:**
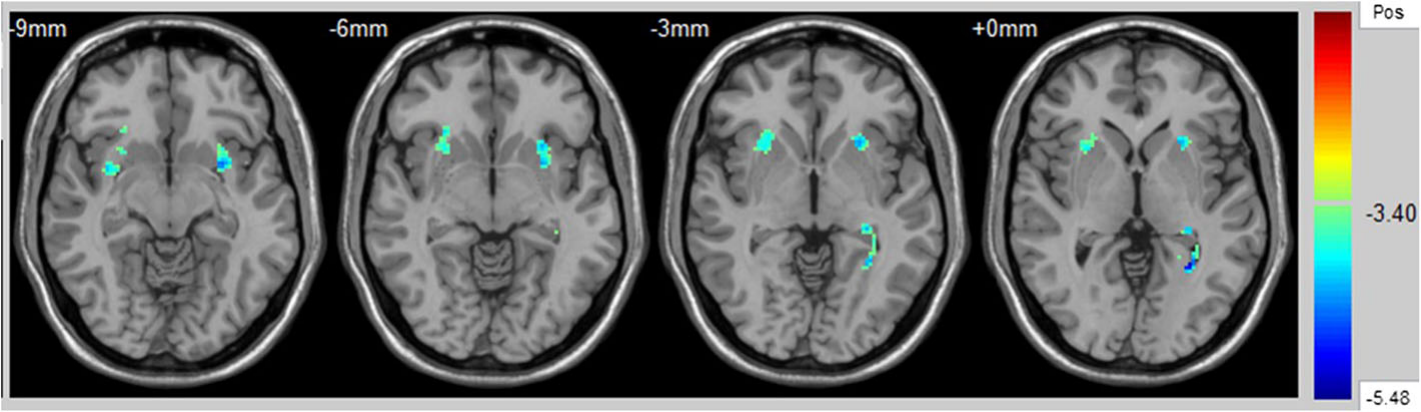
Results of two-sample *t*-tests showing abnormalities in fractional anisotropy (FA) in patients with bipolar disorder (BD) compared with healthy controls aged 26–45 years. The significance of these findings corresponded to a corrected *p* value of < 0.05 (uncorrected *p* value of < 0.001)

FA values did not differ significantly in the UF between the HCs and BD patients aged 13–25 years (right uncinate: *t* = 0.24, *p* = 0.77; left uncinate: *t* = − 0.05, *p* = 0.96). However, the FA values differed significantly in the UF between HCs and BD patients aged 26–45 years. These findings corresponded to a corrected *p* < 0.05 (uncorrected *p* < 0.001) (Fig. 2). Compared with HCs, the BD group aged 26–45 years had significantly decreased FA values in the UF (right uncinate: *t* = 3.35, *p* = 0.001; left uncinate: *t* = 3.40, *p* = 0.001).

Additional exploratory ANCOVA analyses (or two-sample *t* tests) and correlation analyses were performed to determine the effects of states, first-episode status, medication status, and duration on Z values in BD patients aged 13–25 years and FA values in BD patients aged 26–45 years (see Additional file 1). The ANCOVA analysis showed significant differences in the FC of the amygdala-ventral and dorsal PFC in female patients with BD aged 13–25 years. Post hoc analyses showed increased FC in patients in the manic state group compared with those in the stable state group in patients aged 13–25 years (*t* = − 0.11, *p* = 0.01).

## Discussion

In this study, we detected abnormalities in amygdala-PFC FC in patients aged 13 to 25 as well as significantly different FA values in the UF in patients aged 26 to 45 compared with HCs. These findings provide the first evidence of abnormalities in the FC of the amygdala-PFC neural circuit in young female patients with BD and disruptions in the structural integrity of uncinate WM in older female patients in BD. Therefore, the FC of the amygdala-PFC neural circuit may be impaired in female patients with BD during adolescence and young adulthood, while the structural integrity of this neural circuit may be impaired in female patnts with BD during adulthood. We speculate that these findings are significantly related to brain development, female hormone levels, and the course of the disease. When patients with BD are 13–25 years old, the disease may cause serious damage to the function of the brain and no obvious damage to the structural brain because the course of BD is relatively short. However, when the patients are 26–45 years, the brain is mature while the female endocrine system is stabilizing, resulting in brain function that is relatively perfect. Thus, although the damage to brain function is not obvious because of the improvements in and compensation of the brain’s functional system, the damage to brain structure becomes significant with longer duration of the disease. Additional exploratory ANCOVA analyses (or two-sample t tests) and correlation analyses were performed to determine the effects of clinical characteristics on FC/FA in each age group. The results suggested that only the state affected the FC of the amygdala-ventral and dorsal PFC circuitry between manic and stable groups in female patients with BD aged 13–25 years. This requires further research to explore the effects of state on disease.

The amygdale-PFC neural circuit appears to be an important component of brain development during adolescence [47, 48]. Our results indicated that female patients with BD had abnormalities in the FC of the amygdala-PFC circuit during adolescence and young adulthood. Previous studies have also reported that adolescents and young adults with BD exhibit abnormalities in the amygdala and PFC and in amygdala-PFC function [49–52]. However, sex differences were not examined in those studies. For example, in the rs, youth with BD have abnormal connections in the network between the amygdala and regions that are critical for emotional processing and self-awareness [49]. FC in the ventral anterior cingulate and orbitofrontal cortices has been shown to be decreased in an adolescent BD group compared to a HC group during the processing of emotional faces [48]. These findings suggest that the effects of the FC of prefrontal regions and the amygdala are decreased in working memory networks in pediatric BD. Garrett et al. [52] have suggested with their fMRI results that the PFC regulation of heightened amygdala responses to emotional stimuli is deficient in pediatric patients with BD. Additionally, abnormalities in the volumes of the amygdala [53] and PFC [8, 54] have been shown in adolescents and young adults with BD. Therefore, we have speculated that the changes in this neural circuit may be related to the pathophysiology of BD in females during adolescence and young adulthood. Additional support for our speculation has been shown in offspring (mean age = 13.8 years) of parents with BD who exhibit altered amygdala-PFC responses to facial emotion [19]. However, our results did not show any change in rs FC from the amygdala to the PFC in female patients with BD aged 26 to 45. In the adult female patients, the function of the amygdala-PFC neural circuitry was not altered, which was not consistent the results of other studies of male and female subjects with BD. FC with a low frequency between the ventral PFC and amygdala during the rs has been reported as abnormal in adults with BD [22]. Additional studies have reported a dysfunctional connection in the prefrontal-amygdala circuitry in adults with BD [25, 28]. Adults with BD exhibit increased amygdala-medial PFC connectivity and decreased connectivity between the amygdala and dorsolateral PFC [23]. The studies that have included male subjects may have contributed to the differences in the findings compared with those of our study. Thus, the inconsistent results between our study and previous studies were probably due to the inclusion of only women in our study, and it may be due to differences in sex. Future studies are needed to further investigate this issue.

Interestingly, our results indicated significant differences in the FA values in the UF in female patients with BD aged 26–45 years, suggesting that the structural integrity of this neural circuit is impaired in female patients with BD during adulthood. This is consistent with the results of previous structural studies that included both adult men and women and reported significantly decreased FA in BD patients during adulthood compared with the control group [9, 34, 36, 37, 55]. In contrast, other studies have shown that adult patients with BD exhibit significantly increased FA compared with healthy controls in the left UF [32, 33]. The reason for this difference may also be due to our study only including female participants and the differences in sex. This also requires future investigations. However, our results did not indicate any changes in FA in the UF in female patients with BD aged 13–25 years, thus suggested that BD during adolescence and young adulthood in the females did not damage the structure of the UF. However, this is not consistent with the results of a previous study of both male and female subjects that reported FA changes in the UF of adolescents with BD [56]. Taking all of the results into account, we speculate that the structural change in this neural circuit may be related to the pathophysiology of BD in females during adulthood.

A number of limitations should be noted in this study. First, this is a cross-sectional, not a longitudinal, study; therefore, any conclusions must be confirmed by future research. Second, we did not detect significant main effects of mood states on FC and structural integrity. Third, this study lacked the socioeconomic information, which must be supplemented in future. Fourth, we only investigated abnormalities of the functional and structural neural circuitry in females with BD, and we did not study males with BD. Additionally, our relatively small sample size may limit the generalization of our results. Future studies with a larger sample size that includes males of different ages will be important to further understand the neuropathophysiology of BD.

## Conclusion

Our findings by a cross-sectional study have provided evidence that alterations in functional and structural brain development may occur at different age stages in female patients with BD. During adolescence and young adulthood, abnormalities were observed in the FC of the amygdala-PFC neural circuit, while the structural integrity of this neural circuit was altered during adulthood. These findings may be associated with the pathophysiology of BD in females.

## Additional file

Additional file 1: Relationship between FC of amydala-PFC or FA and clinical characteristics in the female BD group aged 13 to 25 or aged 26 to 45. (DOC 43 kb)

## Abbreviations

BD: Bipolar disorder
BPRS: Brief Psychiatric Rating Scale
DTI: Diffusion tensor imaging
FA: Fractional anisotropy
HAMA: Hamilton Anxiety Rating Scale
HAMD: Hamilton Depression Rating Scale
HC: Healthy controls
PFC: Prefrontal cortex
rs-fMRI: Resting-state functional magnetic resonance imaging
SCID: Structured Clinical Interview for DSM-IV Axis I Disorders
UF: Uncinate fasciculus
WM: White matter
