# Supplementary material for: Age-specific effects of structural and functional connectivity in prefrontal-amygdala circuitry in women with bipolar disorder
Source: BMC Psychiatry. 2018 Jun 5;18:177. doi: 10.1186/s12888-018-1732-9 (PMC5989351; doi:10.1186/s12888-018-1732-9)
Supplement: Supplementary file 1 — Relationship between FC of amydala-PFC or FA and clinical characteristics in the female BD group aged 13 to 25 or aged 26 to 45. (DOC 43 kb) [file 12888_2018_1732_MOESM1_ESM.doc]

**Additional file 1**

**Relationship between FC of amydala-PFC or FA and clinical characteristics in the female BD group aged 13 to 25 or aged 26 to 45**

We extracted Z values from the gray matter mask in female patients with BD aged 13–25 years and FA values from the white matter mask in the BD group aged 26–45 years. Additional exploratory ANCOVA (or two-sample *t*-tests) and correlational analyses were performed to determine the effects of state, first-episode status, medication status, and duration on the Z values of the BD patients aged 13–25 years and FA values of the BD patients aged 26–45 years (Table S1/S2). The ANCOVA results showed significant differences in FC of the amygdala-ventral and dorsal PFC in the female patients with BD aged 13–25 years among the 3 state groups. Post hoc analyses showed higher FC of the amygdala-ventral and dorsal PFC in the manic state group compared with those in the stable state group in BD patients aged 13–25 years (t = -0.11, *p* = 0.01). The results suggested that only the state affected the FC of the amygdala-ventral and dorsal PFC circuitry between manic and stable groups in female patients with BD aged 13–25 years. This requires further research to explore the effects of state on disease.

**Table S1. Relationship between FC of amydala-PFC and clinical characteristics in the female BD group aged 13 to 25**

|  | **CL1** | |  | **CL2** | |  | **CL3** | |  | **CL4** | |
| --- | --- | --- | --- | --- | --- | --- | --- | --- | --- | --- | --- |
| **clinical characteristics** | F/T/R values | P values |  | F/T/R values | P values |  | F/T/R values | P values |  | F/T/R values | P values |
| State (depressed/manic/stable ) | 3.47 | 0.04 |  | 0.53 | 0.59 |  | 1.31 | 0.28 |  | 0.69 | 0.50 |
| First episode, yes | 0.33 | 0.74 |  | -0.046 | 0.96 |  | 0.72 | 0.47 |  | 0.78 | 0.44 |
| Medication, yes | 0.99 | 0.32 |  | -0.21 | 0.83 |  | 0.81 | 0.42 |  | 1.12 | 0.27 |
| Duration (month) | 0.07 | 0.63 |  | -0.02 | 0.92 |  | 0.01 | 0.93 |  | -0.09 | 0.53 |

CL, cluster; CL1, Ventral and dorsal prefrontal cortex; CL2, Ventral prefrontal cortex; CL3, Dorsal lateral prefrontal cortex; CL4, Dorsal lateral prefrontal cortex.

**Table S2. Relationship between FA and clinical characteristics in the female BD group aged 26 to 45**

|  | **Right uncinate fasciculus** | |  | **Left uncinate fasciculus** | |
| --- | --- | --- | --- | --- | --- |
| **clinical characteristics** | F/T/R values | P values |  | F/T/R values | P values |
| State (depressed, manic and stable ) | 1.03 | 0.37 |  | 1.10 | 0.34 |
| First episode, yes | -1.49 | 0.15 |  | -0.49 | 0.63 |
| Medication, yes | 0.73 | 0.47 |  | 0.46 | 0.65 |
| Duration (month) | 0.001 | 0.99 |  | 0.049 | 0.77 |
